# Determinants of Adherence to a Ketogenic Diet in Patients with Heart Failure with Reduced Ejection Fraction

**DOI:** 10.3390/nu18121857

**Published:** 2026-06-09

**Authors:** Lee Patricia Liao, Barbara Murphy, Gary C. H. Gan, Liza Thomas, Luigi Fontana, Shannon McKinn, Sarah Zaman

**Affiliations:** 1 Westmead Applied Research Centre, Faculty of Medicine and Health, University of Sydney, Sydney 2145, Australia; lee.liao@sydney.edu.au (L.P.L.); liza.thomas@sydney.edu.au (L.T.); 2 Australian Centre for Heart Health, Melbourne 3051, Australia; barbara.murphy@australianhearthealth.org.au; 3 Melbourne School of Psychological Sciences, The University of Melbourne, Melbourne 3010, Australia; 4 Department of Cardiology, Westmead Hospital, Sydney 2145, Australia; chgan84@gmail.com; 5 Department of Cardiology, Blacktown Hospital, Sydney 2148, Australia; 6 South West Clinical School, University of NSW, Sydney 2170, Australia; 7 Charles Perkins Centre, Faculty of Medicine and Health, University of Sydney, Sydney 2006, Australia; luigi.fontana@sydney.edu.au; 8 Department of Endocrinology, Royal Prince Alfred Hospital, Sydney 2050, Australia; 9 School of Public Health, University of Sydney, Sydney 2006, Australia; shannon.mckinn@sydney.edu.au

**Keywords:** heart failure, ketogenic diet, qualitative, adherence, dietary patterns, facilitators, barriers

## Abstract

**Background:** Dietary interventions in heart failure (HF) remain limited, with current guidance focused largely on sodium restriction. Ketone metabolism has emerged as a potential therapeutic target in HF, with ketone supplementation shown to improve cardiac function. However, there are currently no studies investigating factors affecting adherence to a ketogenic diet (KD) in HF. **Aim:** To explore the factors influencing adherence to a KD in patients with HF to inform future dietary interventions. **Method:** This qualitative study was embedded within the KETO-HF pilot randomised controlled trial, in which participants with HF with reduced ejection fraction undertook a 4-month KD. Consenting participants were invited to complete semi-structured interviews. Interviews were audio-recorded, deidentified and transcribed verbatim. Data were analysed using thematic analysis with a mixed inductive–deductive strategy. **Results:** Fifteen participants were interviewed. Facilitators of adherence were: (1) personal motivation and self-regulation; (2) improved well-being; (3) interpersonal support and; (4) adaptive strategies and improved nutritional literacy. Barriers included: (1) early-phase physiological and psychological challenges; (2) social and cultural friction; (3) competing family and work demands and; (4) limited availability of suitable foods, particularly difficulty managing social situations and dining out. **Conclusions:** Adherence to a KD in people with HF is shaped by a combination of individual and social factors. These findings highlight the need for improved education, support, and increased food options to optimise implementation of dietary ketosis in HF.

## 1. Introduction

Heart failure (HF) affects an estimated 56 million people worldwide [1]. In Australia alone, HF caused 5000 deaths in 2022 [2], and HF-related hospital admissions generate an estimated $600 million in direct costs annually [3]. Heart failure with reduced ejection fraction (HFrEF), defined by a left ventricular ejection fraction (LVEF) of ≤40%, is a chronic, progressive condition and a leading cause of hospitalisations worldwide [4]. Despite advances in guideline-directed medical therapy, individuals with HFrEF continue to experience markedly reduced survival, with life expectancy remaining up to 60% lower than that of the general population [5].

A ketogenic diet (KD), characterised by very low carbohydrate intake (20–50 g/day; 5–10% daily intake) [6], high fat (70% daily intake) and moderate protein (20% daily intake) consumption, is designed to induce nutritional ketosis. Owing to its anti-inflammatory and weight loss benefits, a KD has been proposed as a beneficial eating pattern in cardiometabolic conditions [7]. In animal models of HF specifically, circulating ketones may provide an additional fuel source for the failing myocardium [8]. In fact, the failing heart’s metabolic substrate shifts from glucose to ketone bodies, the latter of which produces more energy than glucose [9]. In people with HF, a meta-analysis of six studies assessing the effects of KD in HF (n = 99) demonstrated that short-term ketone supplementation (exogenous intake of a ketone supplement orally or via infusion) leads to improvements in left ventricular ejection fraction (LVEF) [10]. A short-term KD has also been shown to improve haemodynamic and metabolic parameters in HF patients (n = 377), which remained stable after 1 year [11]. There is caution against a KD in patients with ischaemic-related HF, due to potential increases in LDL cholesterol, known to promote atherosclerosis [12]. Furthermore, despite short-term benefits, concerns remain regarding the suitability of a KD for people with HF, particularly given the high burden of comorbidities such as diabetes coupled with a sustained low-carbohydrate intake, and the risk of nutritional inadequacy. These issues become especially salient in later stages of HF, when patients are more vulnerable to cachexia, sarcopenia and potential interactions with complex medication regimens.

For any dietary intervention to achieve meaningful and sustained clinical benefit, adherence is critical. However, there are currently no studies examining adherence to a KD in people with cardiovascular disease, including those with HF. Adherence is influenced by a complex interplay of intrinsic and extrinsic influences that shape an individual’s ability to maintain long-term behavioural change. Most previous studies on KD adherence involve patients with epilepsy, diabetes and obesity [13,14,15,16]. In studies involving patients with type 2 diabetes mellitus, adherence to a KD or low-carbohydrate diet has been shown to vary ranging from 10% (at 16 weeks) to 96% (at 1 year), although this was dependent on the method of measuring adherence [17,18]. Perceived improvements in clinical outcomes such as discontinuing or reduction in medication usage [18] served as a key motivators for sustaining dietary adherence while limited support from healthcare providers and insufficient resources and information were major barriers to long-term adherence [14,16]. Similarly, in a 16-week low-carbohydrate intervention for patients with diabetic cardiomyopathy, many participants expressed disappointment at the lack of nutritional guidance they received [15]. Other challenges in adhering to low-carbohydrate diets relate to difficulties with dietary restriction, social and cultural pressures, food availability, and sustaining long-term behavioural change [19,20]. These challenges may be further compounded in people with HF as managing HF is already inherently complex, so adherence to dietary recommendations is often compromised by factors such as poor motivation and limited understanding of the benefits of regular exercise, alongside the need to incorporate multiple pharmacological therapies [21]. Physical factors such as fatigue and reduced functional capacity, and social factors such as the availability of emotional, informational, and practical support from family, friends and healthcare providers, might also compromise adherence in people with HF [22]. These overlapping demands highlight how challenging sustained adherence can be in the context of HF. Together, these findings highlight the importance of understanding the barriers that patients face so that clinicians can better support them in adopting and sustaining these dietary approaches. The lack of studies in adherence to a KD thus limits current understanding of the barriers to adherence in this population of HF patients.

The present study aimed to explore the factors influencing adherence to the KD among patients with HF. This was with the overall aim of determining key motivators and barriers to inform the design of future KD interventions.

## 2. Materials and Methods

This qualitative study was conducted as a sub-study within KETO-HF trial, an investigator-initiated, open-label, prospective pilot randomised controlled trial (RCT) approved by the Western Sydney Local Health District Human Research Ethics Committee (2023_ETH00989). The parent trial will be enrolling 60 patients (recruitment since 29 January 2024 and ongoing) with HFrEF (n = 30 in each arm) who are randomly assigned in a 1:1 ratio to either the control (no dietary intervention) or the intervention group receiving a normocaloric KD for a duration of 4 months. After randomisation, a one-on-one dietician consultation provided detailed advice (oral and written; see Supplementary S2) on a KD emphasising 20–30 g/day of carbohydrate (<5–10% daily intake), high healthy fat (70% daily intake) and moderate protein (20% daily intake) consumption. Instruction was provided on the use of a food diary and/or smart phone app to record carbohydrate intake per day (g/day) and performing urine ketone measurements at home. Follow-up visits occurred at 1 week, 1 month, 3 month and 4 months, where study coordinators reviewed dietary records and home blood/urine ketone measurements to assess compliance. Dietitian consultations also occurred at these follow-up time points. * For full description of the selection criteria and intervention, see the Australian and New Zealand Clinical Trials Registry (ACTRN12624000058572, 25 January 2024).

### 2.1. Study Design

We employed an interpretive, phenomenological methodology using both an inductive and deductive thematic analysis approach. The phenomenological approach enabled us to explore the core meaning of participants’ experiences, including how they interpreted and made sense of dietary change and the ways it shaped their everyday lives. This involved one to one semi-structured interviews (as detailed below) where interview questions were framed by two theoretical models—primarily, the Health Belief Model (HBM) and the Theory of Planned Behaviour (TPB) (Table 1). Employing the HBM enabled the examination of health behaviours through individuals’ perceptions and beliefs. The second framework, TPB, was selected to gain an understanding of the influence of subjective norms (i.e., support networks) and planned behavioural control.

### 2.2. Participants

Potential participants were identified through screening of individuals currently attending or previously seen at HF clinics at two hospitals in Sydney, Australia. These hospitals were both part of the Western Sydney local health district (Blacktown and Westmead Hospital). Recruitment was also be achieved via flyers advertised in cardiology clinics and social media platforms. Patient lists from cardiology clinics at Blacktown and Westmead hospital were also screened for eligibility. Patients who were eligible were approached either in-person at the time of their HF clinic attendance or over the telephone. All participants who were eligible and agreed to participate then attended in person to undergo consent, followed by baseline assessment, fasting pathology, echocardiography and randomisation by the study investigator.

Fifteen participants were recruited into the present sub-study, representing 50% of the intervention cohort providing a sufficiently large proportion to capture variation in experiences. Although data saturation was reached by participant 10, we continued to 15 to ensure adequate depth and diversity of perspectives. This reflects a purposive sampling approach, selecting information-rich cases that could best illuminate the research question. All participants were aged 18 years and older, were able to understand the English language and communicate clearly, had a diagnosis of HFrEF (LVEF ≤ 40%) and had been randomised to the intervention arm of the KETO-HF trial. Two participants initiated but did not complete the trial; their data were retained to help identify potential barriers to engaging with and sustaining the intervention.

### 2.3. Data Collection

Interviews were undertaken between 29 January 2025 and 12 May 2026 by the first author (L.P.L.) and took place on the date of their final follow-up of the 4-month intervention. Interviews were conducted in English; duration ranged from 20 to 40 min. The interviewer did not have any prior relationships with the study participants. Interviews were conducted either in person or via Zoom, depending on participant preference and availability. All interviews, including those held face-to-face, were digitally audio-recorded using the Zoom video conferencing platform. Participants were provided with the opportunity to ask questions before the recording commenced. A range of open-ended questions and targeted probing were used throughout the interviews to encourage rich, unrestricted participant engagement (Table 2).

Zoom automatically generated both audio and video recordings and preliminary transcripts using its built-in transcription function. These transcripts were then manually reviewed by the first author and edited for accuracy and coherence and subsequently de-identified prior to coding. After transcription all audio and video recordings were deleted.

### 2.4. Data Analysis

Deductive coding was informed by constructs from the HBM and TPB, which provided an initial set of sensitising concepts—perceived benefits, perceived barriers, attitudes, subjective norms, and perceived behavioural control. These guided the early organisation of the data, while inductive coding allowed us to develop unanticipated themes. We used the 6-step model for reflexive thematic analysis as proposed by Braun and Clarke [25,26]. L.P.L. immersed themselves in the dataset through repeated reading of the transcripts to develop familiarity, being mindful of early impressions (step 1). Then, L.P.L. systematically coded the meaningful features of the data (step 2) and organised these codes into initial themes, supported by a developing thematic map (step 3). L.P.L. reviewed and refined preliminary themes by collapsing overlapping ideas and separating distinct concepts to ensure they reflected the dataset as a whole (step 4). A second researcher (S.M.) independently reviewed the coding and thematic structure. Subsequently, L.P.L. defined and named themes (step 5) to capture their central meaning in discussion with S.M., B.M., and S.Z., and produced a full analytic report (step 6).

## 3. Results

### 3.1. Participant Characteristics

A total of 15 participants consented to be interviewed (12 male and three female). The mean age of participants was 56.9 ± 12.6 years with New York Heart Association (NYHA) HF classification between I and III. Participant demographics are shown in Table 3.

### 3.2. Themes and Sub-Themes Identified

The analysis brought about facilitators and barriers to adherence, comprising eight themes—and a total of 20 codes. Themes identified within the ‘facilitators’ category were: (1) personal motivation and self-regulation; (2) improved well-being; (3) interpersonal support; and (4) adaptive strategies and nutritional literacy improvement. Themes identified within the “barriers” category were: (1) early-phase physiological and psychological challenges; (2) social and cultural friction; (3) family and work demands; and (4) limited food availability (Table 4).

### 3.3. Facilitators to Adherence

#### 3.3.1. Theme 1: Personal Motivation and Self-Regulation

A strong and consistent theme was the role of personal motivation and self-regulation. Participants demonstrated a clear awareness of the seriousness of their condition and this self-understanding played a central role in motivating adherence.

*“I had an enlarged heart and a lot of blocks throughout my body. My heart had taken a beating at that time (…). So I said to myself I’m going to fully commit and its only 3–4 months of your life, you know?”*

(P7, male, 46 years old, NYHA Class II).

Such acknowledgement of the extent of their condition seemed to act as a powerful motivator, reinforcing their commitment to maintaining the dietary regimen.

Participants who viewed their health as something they could actively influence were more driven to sustain the dietary changes.

*“When I started doing the diet, it felt better to eat healthier. When I ate my old diet, you feel happy and content, but then the after-effects are not good, because you feel lethargic. Whereas, when I keep on this diet, I feel full and have more energy.”*

(P6, male, 42 years old, NYHA Class II).

Self-control and critical insight were demonstrated when participants reflected on their own behaviours, particularly when thinking about how they managed to maintain the diet.

*“I found myself occasionally going to Red Rooster and just get quarter of a chicken but resist eating the chips.”*

(P12, male, 55 years old, NYHA Class I)

This ability to recognise tempting situations and consciously resist them indicates that motivation played a central role in sustaining adherence, with participants deliberately engaging in behaviours that supported their goals.

There was a determination to see change and a willingness to face the interventional challenge. Participants explained that they needed to shift their mindset and adopt a more disciplined approach to their daily habits, including pushing themselves to be more active and engaged in daily responsibilities such as work. This shift was often framed as a contest between their heart condition and their broader health, with a strong desire to “win”.

*“I couldn’t mow the lawn and I thought I was going to be semi-retired at my age (…) then I started to become more aware and then I thought, there is no way, I’m not going to accept this. That’s it! I’m just going to stick to this diet and also get active.”*

(P8, female, 58 years old, NYHA Class II)

#### 3.3.2. Theme 2: Improved Well-Being

A marked improvement in well-being was spoken about extensively by participants as they described their surge in energy levels as they progressed throughout the intervention. They reported a substantial improvement in clarity of mind and thought which gave them a more positive outlook to life.

*“My quality of life became better and all through the month I was happier (…) then I would keep going on the diet for longer periods and I taught myself the discipline to continue”*

(P6, male, 42 years old, NYHA Class II).

Participants spoke about being able to resume everyday activities and feeling re-energised in their daily lives. Observable indicators of progress such as weight loss and reductions in fatigue acted as powerful motivators, encouraging patients to adhere even more closely to the diet.

*“I mean the sort of 3:30 itis, I found to be significantly reduced. But then, you know the benefit of keeping to the diet was the weight just melting off really was fantastic! and that was like, Oh, geez! And all of a sudden, you know, 96, 95, 94, 93 kg. Then I’m dipping below 90 and into the 88. And I’m like, Wow.”*

(P2, male, 44 years old, NYHA Class I)

Likewise, improved body image was a motivator for adherence to the diet.

*“One top that I bought never fit me (…) And then, as I was going through the keto diet I thought, I’ll try this top on, and found that it fits me nicely.”*

(P8, female, 58 years old, NYHA Class II)

#### 3.3.3. Theme 3: Interpersonal Support

Having the presence of support networks including family, friends and healthcare providers was a critical factor for sustained dietary maintenance.

*“My mum and my daughter were very, very good and always kept food which I could eat in the fridge (…) they had special meal for me and everyone else’s on the side”*

(P7, male, 46 years old, NYHA Class II)

*“The whole healthcare team were a great support the whole time and I didn’t find one personality that was hard to deal with, or that you had to persevere with.”*

(P11, male, 72 years old, NYHA Class II)

Notably, two forms of support consistently surfaced as central to making the intervention feel more accessible for participants. When a significant other was simultaneously undertaking some form of dietary change, participants felt like they had a companion also undergoing a challenge.

*“My husband is on a diet to eat more healthy (…) and he’s eating more protein (…) so that’s encouraging for me to see.”*

(P8, female, 58 years old, NYHA Class II)

Participants also described another form of support where another individual would actively keep them accountable.

*“My wife joined me in the beginning because she wants me to do it as well and said it will make it easier for me. She said, we can do it together (…) so yeah, initially she was my guide.”*

(P6, male, 42 years old, NYHA Class II).

Together, these accounts demonstrate how interpersonal support substantially reduced the practical and emotional burden of the diet, enabling participants to feel more confident and sustain adherence over time.

The dietitian was singled out as one of the key people in the healthcare team pivotal to participant success. Specifically, the dietitian helped integrate the new eating pattern into participants’ routine with minimal disruption to their daily activities, which made the changes feel more feasible. Moreover, the follow-up calls from the team also helped keep participants accountable.

*“If I hadn’t had the support of the dietitian, I couldn’t have done it (…) her advice on food substitutes was really useful and she explained to me to replace bad fats with good fats. Having a dietitian makes a big difference”*

(P9, female, 60 years old, NYHA Class I)

#### 3.3.4. Theme 4: Adaptive Strategies and Improved Nutritional Literacy

A prominent theme was willingness to adapt when participants found new substitutes they had not previously known about or when they discovered a new recipe with food substitutes. Participants expressed how much easier it was to manage the diet once they were familiar with all the substitutes.

*“A brand called Noshu has got a low carb pancake mix and a brownie mix. You have one little pancake and that’s only 7 g carbs and it meant for my birthday I could still have cake which was great.”*

(P2, male, 44 years old, NYHA Class I)

Once participants pushed through the initial period of cravings, they reported that they no longer had an appetite for carbohydrates—particularly bread.

*“I don’t even fancy bread anymore. I don’t crave for it. It’s because I’ve conditioned my body to not need it.”*

(P5, male, 68 years old, NYHA Class II)

This shift in appetite functioned as a facilitator, as the reduction in cravings made it easier for participants to maintain the dietary requirements over time.

Furthermore, participants who developed strategies to manage challenges—such as identifying suitable “on-the-go” foods they could eat at work—found it easier to maintain the diet.

*“I was out and saw that the vending machine had beef jerky which I was certain I could have, so I knew the diet had become ingrained in my mind.”*

(P13, male, 38 years old, NYHA Class III)

Those who typically prepared home-cooked meals found it easier to adapt to the diet as they could control the ingredients being added.

*“It helped that I pretty much do the cooking at home (…) I’d do an egg omelet for myself, and then cook a non-egg omelet for her, when making frittatas and stuff, so I was just making two different types of meals, but with two different protein sources”*

(P12, male, 55 years old, NYHA Class I)

Overall, participants described an enhancement in their nutritional literacy which was a goal many had from the start of the intervention. This included differentiating between the type of carbohydrates, understanding nutrition labels and appropriate food alternatives. They also emphasised their desire to deepen their understanding, viewing increased knowledge as a pathway to improving their health.

*“I didn’t realise that by eliminating carbs in food, made me aware of sugar that’s hidden in many foods.”*

(P9, female, 60 years old, NYHA Class I).

### 3.4. Barriers to Adherence

#### 3.4.1. Theme 1: Early-Phase Physiological and Psychological Challenges

Factors surrounding the diet itself contributed to whether participants adhered to the diet consistently. A common experience was the “first-week hurdles” which included cravings, experiencing fatigue, nausea or dizziness, and difficulties getting into a different “food” routine.

*“The 1st week or so was pretty brutal.”*

(P2, male, 44 years old, NYHA Class I)

*“When I started the diet, I felt dizzy and had less energy.”*

(P4, male, 67 years old, did not complete the intervention, NYHA Class I)

Having to push through this period was intense, particularly with the restriction of carbohydrates which regularly accompanied meals. The experience of seeing food was described as “hypnotising” and smells were described as being very enticing. Cravings pressed heavily on participants’ minds, making this phase particularly psychologically difficult.

*“It was very hard from the beginning. I see ice cream, I see soft drink and I see bread and you want it (…) my weakness was soft drink because I craved the sugar.”*

(P1, male, 65 years old, NYHA Class II)

Those who relied heavily on takeaway foods struggled with temptations for carbohydrate-rich options such as potato chips and burgers, particularly when they were surrounded by others eating these foods.

*“You have to watch people eat chips, because every meal got served with chips. I felt like, hiding under the table.”*

(P12, male, 55 years old, NYHA Class I)

#### 3.4.2. Theme 2: Social and Cultural Friction

A major theme reflecting challenges faced by participants was the awkwardness of dining out with work colleagues or friends. They describe the struggle as feeling embarrassed, left out, and unable to eat in a way that feels “normal” to them.

*“It’s just that we spend a lot of time with friends, and all of our friends are good, hospitable people, which means alcohol and all sorts of stuff on the weekends. But with this diet you can’t have the fish and chips on a Friday night with everyone. No potatoes.”*

(P3, male, 79 years old, NYHA Class I)

Cultural barriers were also evident particularly in participants of Asian or Pacific Islander background where the main staple for each meal is rice and dishes are shared.

*“Like in a Filipino diet or Asian diet, you cook a big batch of rice and you eat it with everything.”*

(P6, male, 42 years old, NYHA Class II)

*“I’m Asian and we like to share food so its hard not to eat the noodles or the rice with the shared dishes.”*

(P15, female, 65 years old, NYHA Class II)

Participants from various cultural backgrounds explained that it is considered impolite to eat only prepared dishes without also eating the accompanying rice.

*“Like being Polynesian, we have to eat anything on our plate. But now I have to think about it.”*

(P8, female, 58 years old, NYHA Class II)

For some, social awkwardness was exacerbated due to food availability and choices particularly at restaurants from other cultural backgrounds.

*“Let’s just say you go to a Middle Eastern restaurant or a cuisine that you’re not familiar with and you have no idea what to choose.”*

(P2, male, 44 years old, NYHA Class I)

These situations highlight the need for dietary approaches that accommodate individual priorities, as this ultimately shape what people can realistically sustain in everyday life.

#### 3.4.3. Theme 3: Family and Work Demands

Disruptions to family life were identified as a common challenge. Preparation of food was a significant issue particularly with participants who had families with school-aged children. This meant that different meals had to be prepared for the participant as well as the rest of the family. This was identified as a significant hurdle to their progression.

*“Going into it was a bit harder because of kids. I look after everyone’s food at home.”*

(P6, male, 42 years old, NYHA Class II)

The intervention was described as a psychological strain at times due to life stressors that constrained participants’ ability to enact change, at times overwhelming their sense of agency.

*“It was more the schedule with the family, and just with the hours with work (…). the main reason I had to stop the diet was that I had a few things happen with the family with just a few deaths (…) and it was just that there was too much going on.”*

(P10, male, 40 years old, did not complete the intervention, NYHA Class II)

Participants expressed the obstacle of needing to work whilst being part of the intervention. Particularly for those who worked away from home, there were challenges with the inconvenience of food preparation and practicalities associated with the type of work.

*“When I go on the ship [for work] and we go away then I don’t carry food and I can’t tell work what I need to eat.”*

(P4, male, 67 years old, did not complete the intervention, NYHA Class I)

#### 3.4.4. Theme 4: Limited Food Availability

Challenges with logistics of the diet, namely finding the right foods and places that supplied them, were common. Participants shared the experience of finding the suggested food alternatives restrictive, largely because they were unaccustomed to being told exactly what to eat. Even though they were given an extensive list of options from the dietitian, there was still a sense of overwhelm where it was up to the initiative of the participant to work out the foods included.

*“The dietitian sent me a list of what I can’t eat, and just looking at it, all my favourite stuff was on it like breakfast cereals and bread (…) I only found the low carb breads at the end of the intervention.”*

(P7, male, 46 years old, NYHA Class II).

Participants described how identifying new options required considerable research, which added an extra layer of effort to the process. Shopping for food initially felt arduous, especially when faced with a wide range of products. This difficulty eased once participants became familiar with what they needed to find.

*“Your options are limited (…) then you find that instead of having chips, you have pork crackling, and then instead of chocolate Cherry Ripe, you have a choc cherry protein bar from Aldi.”*

(P2, male, 44 years old, NYHA Class I).

## 4. Discussion

To our knowledge, this is the first study to investigate factors affecting adherence to a KD in people with HF. Participants reported both facilitators and barriers in their attempts to adhere to a KD. Reported facilitators included personal motivation and self-regulation, improvement in well-being, effective support and adaptive strategies, as well as improvement in nutritional literacy. Barriers reported included physiological and psychological challenges early in the intervention, social and cultural friction, demands of life and limited food options available.

The findings suggest that the most influential themes shaping adherence centred on individual-level factors. Participants’ own self-control, motivation, awareness of the seriousness of their condition and determination to see change emerged as central to whether they sustained the dietary changes. This aligns with studies which have shown that intrinsic factors are major drivers for healthy dietary behaviours [27,28]. Furthermore, participants’ strong awareness of the seriousness of their condition aligned closely with the perceived severity construct of the HBM. This self-understanding appeared to function as a core motivational driver, shaping their willingness to adhere to the dietary pattern. Their reflections suggested that recognising the potential consequences of their illness heightened the perceived benefits of adherence and reinforced a sense of personal responsibility for managing their health. Navigating through this early transition phase into the KD required considerable self-control and determination. These individual-level attributes closely mirror the mentalities of self-reflectiveness and responsibility identified by Werner and Risus [27] in their one-year study of motivators for maintaining an alternative diet. Likewise, models of self-regulation in the dietary change process describe experiences as dynamic and highly personalised [29], which parallels with the participants in this study where each person conveyed their own individualised experience. Although these models are typically applied to sustained behaviour change, the present findings indicate that the same dispositions underpinning long-term adherence do shape behaviour during the initial transition phase.

Consistent with other low carbohydrate and KD studies [15,16,30], participants in this study were motivated by early positive outcomes. Participants were motivated when they experienced improved energy levels, well-being and in some cases, weight loss. Wong et. al. [16] found that in patients with Type 1 or 2 diabetes mellitus who followed a KD for at least 3 months, immediate positive results were a strong motivator to continue the diet. Similar to the present study, Morris et. al., [30] found in an 8-week low carbohydrate dietary intervention that patients exhibited improved confidence when they experienced improvements in psychological well-being. Given that many people with HF prioritise day-to-day well-being over longevity of life [31], the improvements in well-being that participants experienced on the KD become a particularly meaningful component of the intervention. These immediate gains align closely with what patients themselves prioritise, reinforcing the diet’s relevance and supporting their sense of self-efficacy during the transition. This rise in self-efficacy that accompanied the benefits experienced by participants is consistent with the TPB, which suggests that when individuals perceive the balance of influences as weighted toward facilitators rather than barriers, sustained behaviour change becomes more achievable [32].

Difficulty navigating social situations and dining out was a common challenge expressed by most participants, aligning with other KD studies due to the restrictive nature of the diet [33,34]. In contrast, the Mediterranean diet, which may also be beneficial for patients with HF, is considerably less restrictive, which may explain why social eating tends to pose fewer barriers for individuals following that pattern [35]. Social and cultural norms are known to affect food selections, and this can result in selections that do not align with dietary recommendations [36,37,38]. The need to avoid carbohydrates in a KD, typically served with most meals, created friction and temptations which challenged the formation of new dietary habits. These experiences highlight that when dietary requirements conflict with what individuals value in their everyday lives—such as shared meals, cultural food practices, and social routines—there is a need for culturally tailored meal plans, restaurant guidance, family-inclusive education, and culturally appropriate carbohydrate substitutes.

Knowing the appropriate food substitutes was identified as the key factor in dealing with these psychosocial challenges. Within the framework of the HBM, participants’ ability to develop practical strategies reduced perceived barriers and strengthened their self-efficacy in maintaining the diet. It was common for participants to express poor nutritional literacy early on; this lack of food choice knowledge resulted in the withdrawal of two participants. This is consistent with epidemiological data which show that approximately 60% of Australian adults have low health literacy [39]. Furthermore, low health literacy, and by extension nutrition literacy, is common amongst HF patients and is associated with poorer outcomes [40] and hence support in this area is necessary. This includes practical, literacy-sensitive strategies such as simplified food lists, visual meal guides, label-reading training, shopping lists, recipes, and early dietitian follow-up. Participants enthusiastically reported improved nutritional literacy post-intervention, demonstrating that the intervention fostered the development of new knowledge.

The presence of solid support networks influences how well a patient can adhere to a new dietary pattern [19] and this was evident in this study. In the context of individuals with HF, this support is even more crucial in a dietary trial, given that they already depend heavily on emotional support and shared experiences to manage the ongoing psychological demands of their condition [41]. Participants identified members of the healthcare team, particularly the dietitian, as pivotal to their success. Aligning with the TPB, this guidance by the dietitian reflects the influence of subjective norms and perceived behavioural control, with professional endorsement both validating the behaviour and enhancing participants’ confidence in their ability to adhere. This one-to-one relationship appears to strengthen individuals’ commitment to maintaining the diet, yet studies suggest that such support is inconsistently available [15,16,42]. Practical strategies to address this gap may include standardised education modules, in-app peer support groups and clear escalation pathways to dietetic support, ensuring that participants receive consistent guidance even when continuous one-to-one contact is not feasible.

The demands of life, including family and work, posed a significant barrier to effective adherence to the KD. In this study, competing life demands of family and work contributed to the withdrawal of two participants from the trial. Participants who needed to prepare meals for children described challenges in meeting the needs of both their families and themselves. Particularly for participants who also had work commitments, time constraints undermined adherence in terms of planning and meal preparation. Lack of time has been reported as a major barrier to the preparation of health meals and maintenance of routines [43], which is in line with the HBM, where these competing demands of daily life functioned as significant perceived barriers. The consequence of time scarcity has been shown to result in fewer home-cooked meals and more take-out foods, which can be detrimental to health [44]. This highlights the need for strategies that simplify meal and food selection, thereby reducing the decision-making burden on participants. Support for family caregivers could include family-centred nutrition coaching and visual meal guides for easier implementation at home.

### Limitations

The present study has limitations that may limit the transferability of the findings. Firstly, while there was heterogeneity in terms of ethnicity and education in the sample, there was an imbalance in gender representation in the sample, with more men than women—only three females out of 15 participants. It is known that there is a higher incidence of HFrEF in men than women [45] which might account for this imbalance, although women are historically less likely to be recruited into lifestyle trials [46]. This may be due to care-giving roles and time constraints, which were evident in this trial. Second, all participants were recruited from a single health district characterised by lower income and education levels and as such, nutrition literacy may differ from that of the broader HF population. Such skewed samples can limit the transferability of qualitative findings because the perspectives of under-represented groups may not be fully captured [47]. Furthermore, the participant cohort consisted of clinically stable, mobile individuals, primarily classified as NYHA I–II, who possessed the capacity to undertake a KD intervention. Consequently, their reported experiences may not reflect those of patients with more severe HF (NYHA III–IV), individuals with heart failure with preserved ejection fraction (HFpEF) presenting different symptomatic and comorbidity patterns. This constraint should be kept in mind when considering how broadly the findings may apply. Lastly, only two participants had not completed the intervention, potentially biassing the findings towards those who were successful at completing the diet thereby further limiting transferability.

## 5. Conclusions

This qualitative study provides insight into the factors that influence adherence to a KD in patients with HF. Participants described a complex interplay of individual, social, cultural, and practical influences surrounding their experience, with individual-level factors being the most influential at shaping adherence to the diet. These findings highlight that adherence to an eating pattern is constrained by the realities of chronic illness, symptom management, daily demands, and the ongoing challenges of adopting a new dietary practice. However, it is important to note that the findings of this study are patient-perceived facilitators and barriers, reflecting participants’ subjective experiences rather than objective clinical outcomes.

The present findings have implications for the design of future clinical trials evaluating KD in HF. For clinicians, the findings highlight the importance of providing practical dietary guidance, anticipating early-phase challenges of dietary change (such as cravings, uncertainty and social pressures), and engaging family members or support networks where appropriate. Importantly, the findings underscore the need for clinicians to recognise the emotional and identity-related aspects of dietary change in HF, and to tailor support accordingly.

Incorporating patient-informed perspectives into trial design will increase the likelihood that a KD can be implemented safely and effectively in this population.

## Figures and Tables

**Table 1:** Definition of two theoretical models, Health Belief Model and Theory of Planned Behaviour, used to frame interview questions.

Theoretical Model	Definition
Health Belief Model	Aims to explain health behaviour through perceptions and beliefs of the individual [23]. This framework consists of the following key components: perceived susceptibility, perceived severity, the perceived benefits of an action, perceived barriers, the cue to action and self-efficacy.
Theory of Planned Behaviour	Intentions to engage in a behaviour can be predicted with considerable accuracy from individuals’ attitudes toward the behaviour, the social norms surrounding it, and their perceived behavioural control. Together, these intentions and perceptions of control explain a substantial proportion of variance in actual behavioural performance [24].

**Table 2:** Interview guide and existing model guiding question construction. HBM, Health Belief Model; TPB, Theory of Planned Behaviour.

Question	Model
Before this intervention, how did you feel about your health, including your heart condition? Did you have concerns about your eating habits? Why?	HBM (*perceived severity: understanding of seriousness and thus their motivation to adhere*)
What did you hope would happen from making changes to your eating pattern as recommended by the intervention? Which of those benefits have you actually experienced or observed?	HBM (*perceived benefits: positive factors which may have encouraged adherence*)
What challenges did you anticipate there would be in following the ketogenic diet? Were there any factors or circumstances that made it easier or harder for you to follow this new eating pattern?	HBM (*perceived barriers: factors which may have impeded adherence*)
What was the reason for which you decided to participate in this study? Was there anything which helped motivate you to stick with the new eating pattern since the intervention has been completed?	HBM (*cues to action: prompts which may have helped maintain the diet*)
Before starting the intervention, how confident were you in your ability to follow the ketogenic diet recommendations? Has your confidence changed after completing the intervention? Prompt: Why or why not?	HBM (*self-efficacy: assesses confidence in maintaining dietary changes*)
Was there someone (family, friend, dietitian, doctor, etc.) who said or did something to help you maintaining the ketogenic diet? Prompt: If so, how did this help you? What kind of support has been most helpful for you when following the diet?	TPB (*subjective norms: evaluating the influence of family and friends and healthcare providers*)
How confident did you feel in managing the requirements for the new eating pattern?	TPB (*perceived behavioural control: assesses patient’s confidence to adhere*)

**Table 3:** Participant demographics and heart failure classification.

Participant Characteristic	
Sample size	15
Age, years, mean (SD)	56.9 (11.9)
BMI	
Healthy	2
Overweight (25–29)	7
Obese	6
Sex	
Males	12
Females	3
Ethnicity	
Caucasian	6
East Asian	3
Southern Asian	2
Maori	2
Pacific Peoples	2
Highest Education Level	
University	4
Vocational	4
High school	7
NYHA class ^1^	
I	5
II	8
III	2
LVEF mean (SD)	36.2 (8.7)
HF aetiology	
Non-ischaemic	11
Ischaemic	4
HF comorbidities	
Hypertension	5
T2DM	4
CKD	1
AF	5

^1^ NYHA, New York Heart Association Functional Classification categorises heart failure into four classes (I–IV) based on severity with I as the lowest and IV as the highest. Abbreviations AF, atrial fibrillation, BMI, body mass index, CKD, chronic kidney disease, heart failure, LVEF, left ventricular ejection fraction, T2DM, type 2 diabetes mellitus.

**Table 4:** Key facilitators and barriers to adherence to a ketogenic diet (KD) in people with HF supported with quotes from participants. Additional quotes are provided in Supplementary Material S1.

Themes	Codes	Quotes
Facilitators		
Personal Motivation and Self–Regulation	Understanding one’s illness Desire for challenge Self–control	“I had an enlarged heart and a lot of blocks throughout my body. My heart had taken a beating at that time (…). So I said to myself I’m going to fully commit and its only 3–4 months of your life, you know?” “When I started doing the diet, it felt better to eat healthier. When I ate my old diet, you feel happy and content, but then the after-effects are not good, because you feel lethargic. Whereas, when I keep on this diet, I feel full and have more energy.” “I found myself occasionally going to Red Rooster and just get quarter of a chicken but resist eating the chips.” “I couldn’t mow the lawn and I thought I was going to be semi-retired at my age (…) then I started to become more aware and then I thought, there is no way, I’m not going to accept this. That’s it! I’m just going to stick to this diet and also get active.”
Improved Well–Being	Weight loss High energy levels	“My quality of life became better and all through the month I was happier (…) then I would keep going on the diet for longer periods and I taught myself the discipline to continue” “I mean the sort of 3:30 itis, I found to be significantly reduced. But then, you know the benefit of keeping to the diet was the weight just melting off really was fantastic! and that was like, Oh, geez! And all of a sudden, you know, 96, 95, 94, 93 kg. Then I’m dipping below 90 and into the 88. And I’m like, Wow.” “One top that I bought never fit me (…) And then, as I was going through the keto diet I thought, I’ll try this top on, and found that it fits me nicely.”
Interpersonal Support	Family and friends as companions Healthcare support	“My mum and my daughter were very, very good and always kept food which I could eat in the fridge (…) they had special meal for me and everyone else’s on the side” “The whole healthcare team were a great support the whole time and I didn’t find one personality that was hard to deal with, or that you had to persevere with.” “My husband is on a diet to eat more healthy (…) and he’s eating more protein (…) so that’s encouraging for me to see.” “My wife joined me in the beginning because she wants me to do it as well and said it will make it easier for me. She said, we can do it together (…) so yeah, initially she was my guide.” “If I hadn’t had the support of the dietitian, I couldn’t have done it (…) her advice on food substitutes was really useful and she explained to me to replace bad fats with good fats. Having a dietitian makes a big difference.”
Adaptive Strategies and Improved Nutrition Literacy	Home-cooked recipes Knowing substitutes Pushing through cravings	“A brand called Noshu has got a low carb pancake mix and a brownie mix. You have one little pancake and that’s only 7 g carbs and it meant for my birthday I could still have cake which was great.” “I don’t even fancy bread anymore. I don’t crave for it. It’s because I’ve conditioned my body to not need it.” “I was out and saw that the vending machine had beef jerky which I was certain I could have, so I knew the diet had become ingrained in my mind.” “It helped that I pretty much do the cooking at home (…) I’d do an egg omelet for myself, and then cook a non-egg omelet for her, when making frittatas and stuff, so I was just making two different types of meals, but with two different protein sources” “I didn’t realise that by eliminating carbs in food, made me aware of sugar that’s hidden in many foods.”
Barriers		
Early Phase Physiological and Psychological Challenges	First-week hurdles Cravings Temptations	“The 1st week or so was pretty brutal.” “When I started the diet, I felt dizzy and had less energy.” “It was very hard from the beginning. I see ice cream, I see soft drink and I see bread and you want it (…) my weakness was soft drink because I craved the sugar.” “You have to watch people eat chips, because every meal got served with chips. I felt like, hiding under the table.”
Social and Cultural Friction	Social awkwardness Cultural differences	“It’s just that we spend a lot of time with friends, and all of our friends are good, hospitable people, which means alcohol and all sorts of stuff on the weekends. But with this diet you can’t have the fish and chips on a Friday night with everyone. No potatoes.” “Like in a Filipino diet or Asian diet, you cook a big batch of rice and you eat it with everything.” “I’m Asian and we like to share food so its hard not to eat the noodles or the rice with the shared dishes.” “Like being Polynesian, we have to eat anything on our plate. But now I have to think about it.” “Let’s just say you go to a Middle Eastern restaurant or a cuisine that you’re not familiar with and you have no idea what to choose.”
Family and Work Demands	Preparation for family Life stressors Demands of work	“Going into it was a bit harder because of kids. I look after everyone’s food at home.” “It was more the schedule with the family, and just with the hours with work (…). the main reason I had to stop the diet was that I had a few things happen with the family with just a few deaths (…) and it was just that there was too much going on.” “When I go on the ship [for work] and we go away then I don’t carry food and I can’t tell work what I need to eat.”
Limited Food Availability	Restrictive food choices Effort of searching for foods	“The dietitian sent me a list of what I can’t eat, and just looking at it, all my favourite stuff was on it like breakfast cereals and bread (…) I only found the low carb breads at the end of the intervention.” “Your options are limited (…) then you find that instead of having chips, you have pork crackling, and then instead of chocolate Cherry Ripe, you have a choc cherry protein bar from Aldi.”

## Data Availability

Data is not publicly available due to privacy reasons associated with the clinical trial. The original contributions presented in this study are included in the article/Supplementary Material. Further inquiries can be directed to the corresponding author.

## Supplementary Materials

The following supporting information can be downloaded at https://www.mdpi.com/article/10.3390/nu18121857/s1, Table S1. Additional quotes for each theme.

## Abbreviations

The following abbreviations are used in this manuscript:

HF	Heart failure
HFrEF	Heart failure with reduced ejection fraction
LVEF	Left ventricular ejection fraction
KD	Ketogenic diet
RCT	Randomised controlled trial
HBM	Health Belief Model
TPB	Theory of Planned Behaviour
NYHA	New York Heart Association
