# Supplementary material for: Determinants of Adherence to a Ketogenic Diet in Patients with Heart Failure with Reduced Ejection Fraction
Source: Nutrients. 2026 Jun 9;18(12):1857. doi: 10.3390/nu18121857 (PMC13304560; doi:10.3390/nu18121857)
Supplement: Supplementary file 1 [file nutrients-18-01857-s001.zip › nutrients-4342667-supplementary.pdf]

**Supplementary Material 1 (S1)**
**Table S1. Additional quotes for each theme**

| Theme component | Quote(s) |
|---|---|
| Facilitator: Theme 1<br>Personal motivation and self-regulation | *"I became so scared after the illness hit me suddenly that I thought well why not try the diet."*<br><br>*".....I nearly died. I thought I have to do something if I am to get better so I decided to do the diet."*<br><br>*".....I had to lose the weight and it was explained to me to cut carbs and that's what really attracted me to the diet."*<br><br>*"When I would go back onto the diet for a longer period, I felt good so it taught me the discipline to maintain the diet and continue"*<br><br>*"So the app would say an orange has 34g of carbohydrates and so I had to delete it from my list of foods I liked to eat."*<br><br>*"I used to eat sweets anytime which is all carbohydrate and I had to resist doing this, change the habit and I just don't do it anymore."* |
| Facilitator: Theme 2<br>Improved well-being | *"The diet is good as I've lost some weight and I sleep better and can physically move around more."*<br><br>*"My weight went from 92 to 70 kg so it kept dropping off which is fantastic."*<br><br>*"The biggest benefit I had out of the diet was that the weight started coming off. So I've lost about 12 kilos."*<br><br>*"You feel low in energy at the start but if you push through it you to start have a lot more energy and feel great"*<br><br>*"My quality of life became better especially the energy levels and so now I'm able to walk around all over the place."* |
| Facilitator: Theme 3<br>Interpersonal Support | *"I have help from my  partner who prepares and cooks all the meals for the diet"*<br><br>*"My wife helped me stay on top of the diet."* |

| | |
|---|---|
| | *"I was guided by the dietitian and.it made the whole process of learning and following the diet so much easier."*<br><br>*"The people that were on the dietary program were really supportive and that helped a lot."*<br><br>*"My son always keeps me accountable, like with foods I could and couldn't eat."*<br><br>*"My son's doing a very high protein diet so he gave me a tips and suggestions of protein bars for the diet."*<br><br>*"The dietitian's advice on the food swapping was a really big help to me."* |
| Facilitator: Theme 4 Adaptive strategies and nutritional literacy | *"I learnt and found good pizza bread bases and muffins which were only 3 grams of carbohydrates per muffin which is good for the diet"*<br><br>*"I didn't like almond milk before but now I do and its something that I can have."*<br><br>*"I'll look at all of the health bars, like the Mayvers and the Noshu keto brands, and whatever's on special, I just throw the bulk in, so I've got enough bars, probably, to last me at least another 2 or 3 weeks."*<br><br>*"I found it hard to resist sweet drinks but I found Kombucha which I could have instead."*<br><br>*"I knew that the diet was ingrained in my mind and I started to learn what I could and couldn't have."*<br><br>*"I found very good substitute keto recipes on the internet for making homemade ice cream."*<br><br>*" I learnt that I was allowed to eat macadamias if I was hungry."*<br><br>*"I started doing research and now I've come up with a whole stack of hacks for food substitutes"*<br><br>*"I got a shock that I couldn't even eat prawns because its got carbohydrates which was an eye-opener."*<br><br>*"The diet has changed my whole focus on sugar, breads and pastas."*<br><br>*"I do all the cooking at home and the shopping which made it easier to follow the diet."* |
| Barrier: Theme 1 Early-phase physiological and psychological challenges | *"It was hard when I missed things early on. I found that hard mentally"* |

| | |
|---|---|
| | *"At first it was hard to try and change my whole diet with all the requirements. I really had to push through and learn everything"*<br><br>*"At first it felt like I couldn't eat and drink what I wanted and could only have certain things."*<br><br>*"At the start, I felt really nauseous and it made me dizzy."*<br><br>*"My daughter found low-carb bread but I didn't want to eat it, otherwise I will crave bread again."*<br><br>*"There is a lot of mental prep and I wish someone had told me before the diet so I was mentally prepared."* |
| Barrier: Theme 2<br>Social and cultural friction | *"It is difficult when you go out with family and have Maccas. You feel left out because you have to eat something else."*<br><br>*"You feel like you have to hide away from all the food when you go out with everyone so that made it awkward."*<br><br>*"When you catch up with family and friends and they like to have a few bevvies and you can't have it which is hard."*<br><br>*" I am Asian so we eat rice and dishes with sauce but when you are on the diet, you can't eat that."*<br><br>*"The way I've been brought up, we eat carbs with meals e.g. potato or rice but you can't have that so it's a change from what we are used to."*<br><br>*"It was hard as we normally eat the dish with chipati, but I would only be able to eat one and I won't be full."* |
| Barrier: Theme 3<br>Competing life demands | *"I started getting a little bit lazy because it was taking too much of my time to do all the family meal preparation".*<br><br>*"It was more the schedule with the family, and just with the hours with work that made it hard to also think about the diet"*<br><br>*"In doing the diet, I had to shift all my meal times and that was hard because of work"*<br><br>*"Helpers in my country cook for me so I could control what I was eating. In Australia, I have to do it myself which is hard with having to work as well."*<br><br>*"During this time, I lost the love of my life which made it harder to focus and deal with the dietary requirements."* |

| | |
|---|---|
| | *"I am on the road a lot so I had to have enough food prepped which takes time."* |
| Barrier Theme 4:<br>Limited Food Availability | *"It was hard keeping the diet when going out because you didn't know what to eat and there wasn't much out there."*<br><br>*"If you want to grab anything from a takeaway place, apart from getting barbecue chicken, your options are extremely limited."*<br><br>*"When you eat out you don't know what they cook with and so you can't eat out much."*<br><br>*"… if I'm out on the soccer field all they have is a steak sandwich and meat pie and you can't have that."*<br><br>*"When I go away on ship then I don't carry food as I eat what is available and they don't have keto foods."*<br><br>*"You've got to know the good sugar options, the Noshu option, the good salt options and things like that."* |

**Supplementary Material 2 (S2)**

Dietary educational materials provided to patients

# What do lower and slower carbohydrate meals look like?

**BBC EVERYDAY GOOD FOOD: LOW CARB COOKING**
Good as a starter just to get ideas; small, cheap and colourful with inspiring pictures. Aimed more at the low carb weight loss market so extra fats readily need to be added to make these recipes more ketogenic. All recipes counted for carbohydrate, protein, fat and calories.

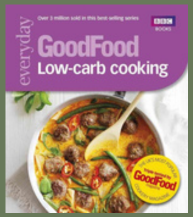


**THE REAL MEAL REVOLUTION AND THE REAL MEAL REVOLUTION LOW-CARB COOKING* (2018)**
Prof Tim Noakes, Jonno Proudfoot* and Sally Ann Creed.
Simple but inspirational with lots of lovely photos.

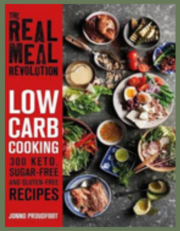


**THE LOW CARB GOURMET - KAREN BARNABY**
Good for grown – up ideas. Lovely recipe ideas.
All recipes counted
for carbohydrate , protein, fat and calories.

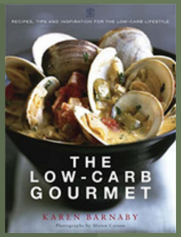


**DIET DOCTOR WEBSITE**
**www.dietdoctor.com**
Lovely clear guidance for starting out on a low carbohydrate lifestyle. Its focus is mainly diabetes and weight management so do be aware of this potential calorie-reduced slant, but the background food guidance is clear, and the visuals are excellent.

https://lowcarbdownunder.com.au/

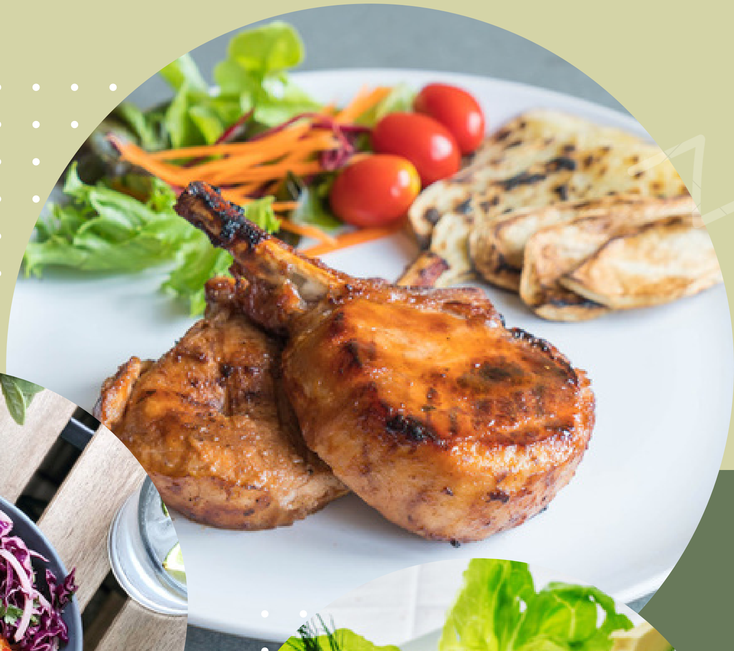

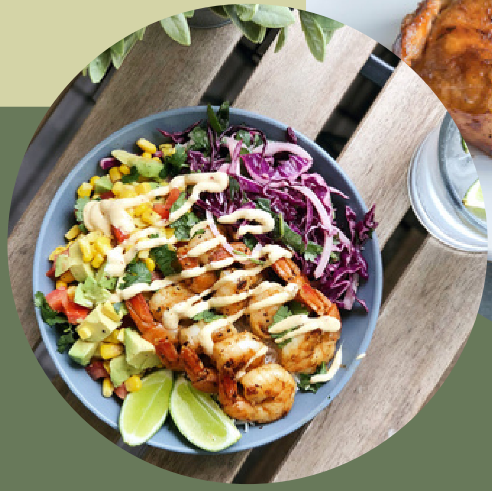

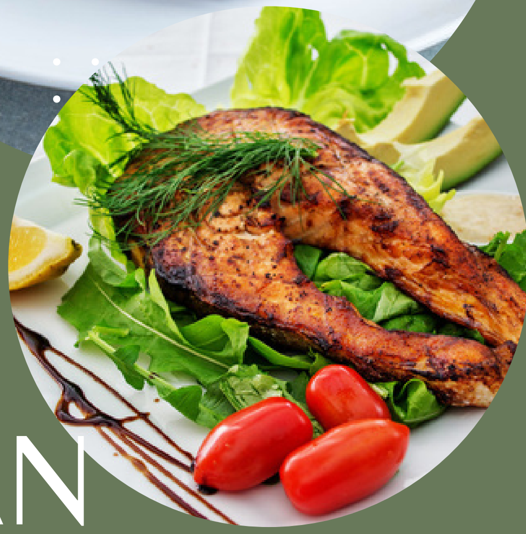

# AN INTRODUCTION TO KETOGENIC THERAPY

# What is a Ketogenic Diet?

A ketogenic diet is a very low carbohydrate, high fat diet designed to encourage the body to switch its main source of fuel from carbohydrate to fat. Ketones are produced as a by-product of this increased “fat burning” process (this is what is meant by ketosis) and the brain quickly adapts to using ketones as the main fuel source for energy production.
All types of diet are based on regular fresh food ingredients; meats, fish, eggs, nuts, seeds, cheese, Olive oil, vegetable oils, butter, cream, vegetables and fruits.

Modified Atkins Diet (or Modified Ketogenic Diet in UK) – not so restricted on protein foods but requires measurement of carbohydrate foods and adequate portions of fats at meals.

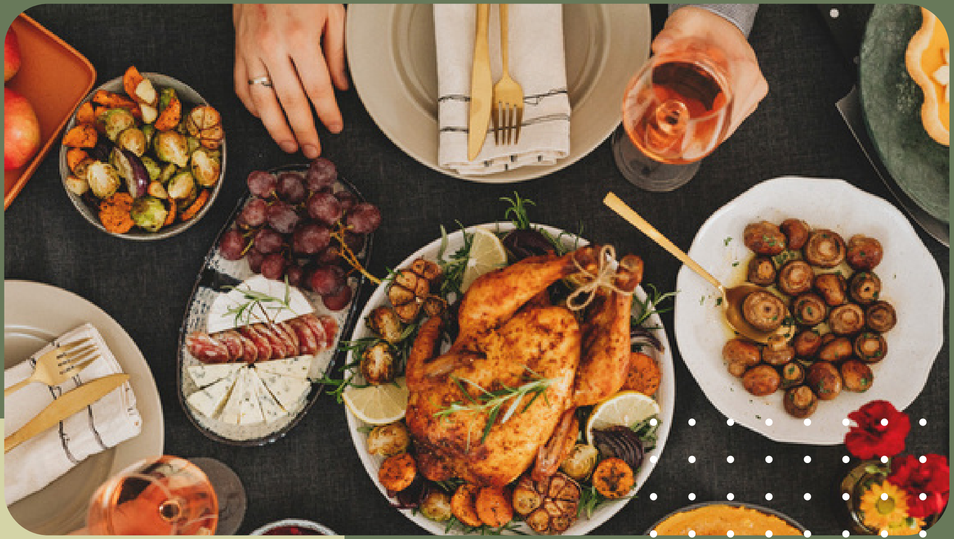

**ketogenic diet is a very low carbohydrate, high fat diet designed to encourage the body to switch its main source of fuel from carbohydrate to fat**

# WHAT’S A MODIFIED KETOGENIC DIET (MKD)?

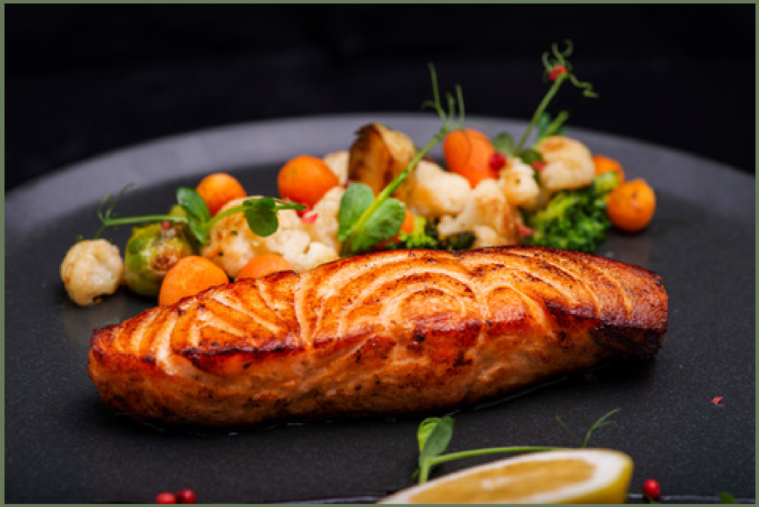

This is based on the Modified Atkins Diet (MAD) evolved by Professor Eric Kossoff and ketogenic team at the Johns Hopkins Hospital in Baltimore USA. At the beginning of the treatment phase you will be asked to limit carbohydrate to 20-30g and you will be given guidance on the amounts of fats you need; with diet adjustments being made based on the changes in your symptoms. Further along in the treatment, MCT oil or MCT supplements can be used as a moderator to enhance ketone production If required.

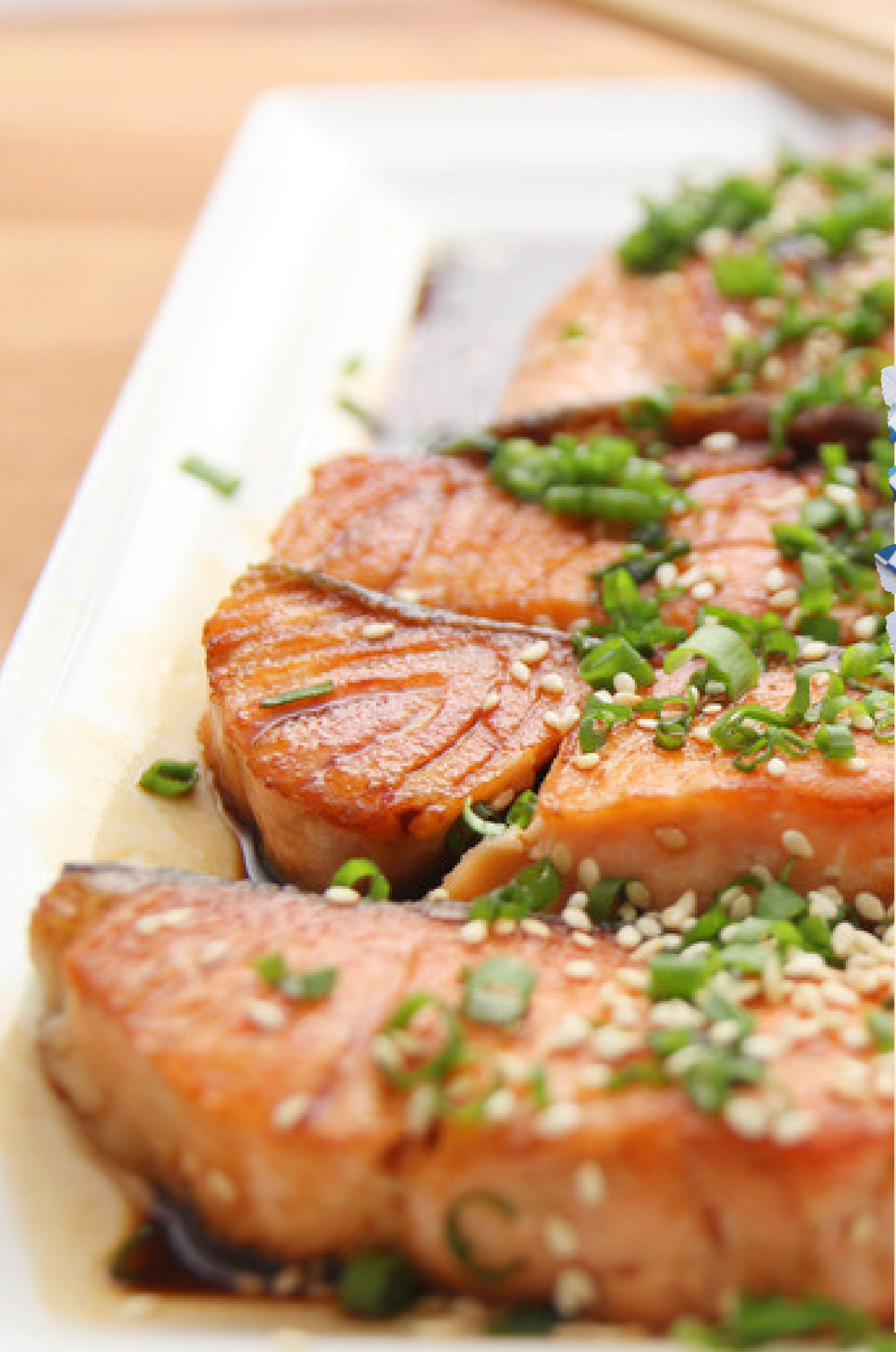

## BREAKFAST TIME

Omelette with mushrooms and tomatoes
Plus berry shake

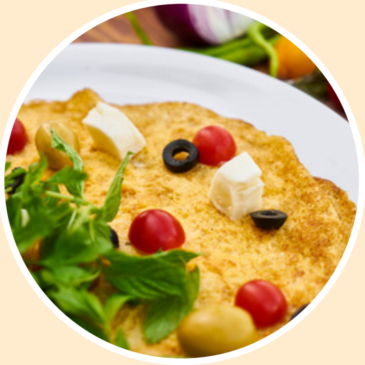

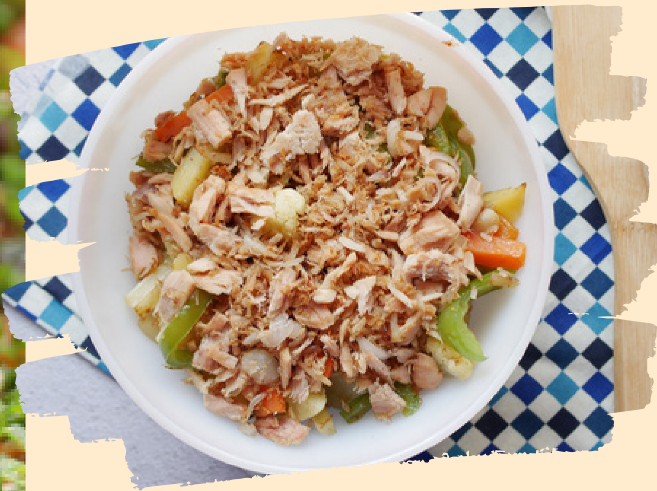

## LUNCH TIME

**Tuna mayo with side salad and citrus dressing**
**Golden flax bread and butter**

## MID-AFTERNOON

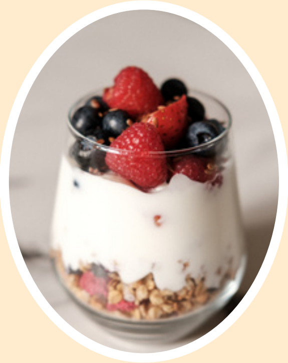

Tea and Snack size Keto Choc muffin

## DINNER TIME

**Chicken spinach and mushroom curry, with cauliflower rice**
**Berries and double cream**

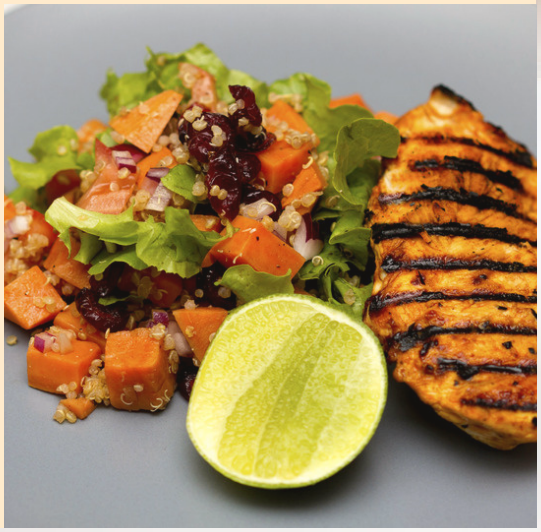

# A considerable change to your eating habits:

A ketogenic diet generally involves a significant shift in food choices and the way your meals look.

## Cooking meals from scratch:

Ketogenic meals generally need to be made from fresh, basic ingredients so a willingness to plan a menu and prepare basic meals is essential. This also means planning meals ahead of time and taking suitable meals and snacks out with you for work, college, travel etc. Eating out becomes easier as you learn more about creating ketogenic meals.

However, we live in a carbohydrate dominant food culture and the availability of keto-friendly meals and snacks is limited,

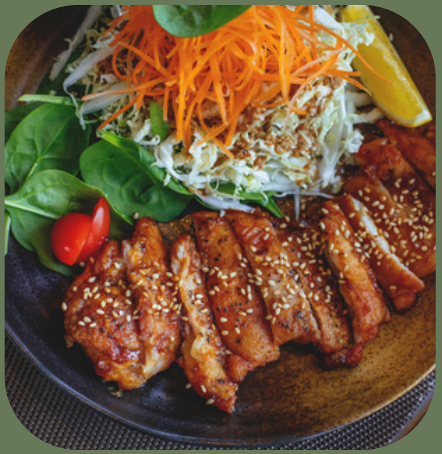

## Careful record keeping:

The value of this can't be emphasised enough. You will need to keep records of your:

- home urine ketone tests ( 3 per week)
- diet and any changes made

## Positive support from friends, family and work colleagues:

A radical changing to your eating habits can be tough under any circumstances but ketogenic therapy is much more than this. You become responsible for delivering your treatment, monitoring the effect and making it work. This can be empowering and worrying in equal measure.

There may be days when you need help with shopping and cooking and so there ideally needs to be at least one other person who understands the principles of your treatment and will work alongside and support you. Moral and practical support from those around you is so important and makes a real difference.

**Ketogenic meals generally need to be made from fresh, basic ingredients so a willingness to plan a menu and prepare basic meals is essential.**

# A summary of the key food changes involved

## Carbohydrate-containing foods; a significant reduction

Carbohydrate control is fundamental to
the ketogenic fuel switch and a therapeutic ketogenic diet for adults will generally contain 20-30g carbohydrates per day. You will be guided on how much to include at meals and how to spread this through the day.

We recommend choosing carbohydrate containing foods that release their glucose more slowly such as non-starchy vegetables, berries, dairy products, nuts and seeds to provide your prescribed amounts at meals. Weight for weight, these foods are also much lower in carbohydrate than traditional starchy sources, so you can
get more food bulk for your carbohydrate allowance. On ketogenic diets, whenever any carbohydrate is eaten, there always needs to be some fat alongside this.

## Fats and oils; a significant change from 'normal'

Fats are the main driver for ketone production and become your main fuel; needing to be included in each meal and snack. Examples of good fat sources are olive oil, coconut oil, butter, lard, double cream, mayonnaise, avocados, nuts and cheese. Protein containing foods such as meats, oily fish and eggs in your meals do naturally provide some fats too, but the amounts are not adequate so extra pure fats need to be added at each meal.

Your prescription will provide you with guidance on how much to use and when. Amounts will depend on your body weight, your day to day activity levels and whether there is a need for weight maintenance or weight reduction.

## Protein containing foods; 'normal' quantities based on appetite

You will be encouraged to include a normal-sized portion of meat or fish or eggs or nuts
or cheese with each meal. On a Modified ketogenic diet, protein foods are not weighed and measured but large portions can deliver much more protein than your body needs, with the excess being burned for fuel. This reduces the need for your body to burn as much fat; impairing ketone production.

## Vitamin, mineral and micronutrient supplements

Baseline vitamin, mineral and trace element supplementation may be recommended alongside ketogenic therapy. This can be provided by a good quality one-a-day A-Z type product aimed at adults. However, very few of these provide calcium, magnesium and Vitamin D in adequate amounts, therefore an additional product may be required.

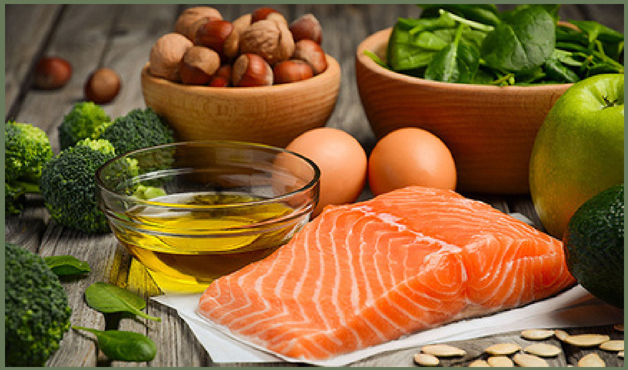

# Are there any side effects?

## During initiation

**As your ketogenic diet has your metabolism takes a few days to adjust and this may lead to lethargy, a mild headache or slight nausea. This is quite normal and should clear after a few days.**
**We advise you to take rest when you need it and time to think about your new food choices and meal preparation.**

## Changes to your digestion

**A ketogenic diet alters your digestive processes and the availability of food and fuel to the millions of bacteria living in your bowel*. The most common side effect associated with this is constipation. This can readily be managed by ensuring adequate fluids and enhancing fibre intake; choosing large portions of very low carbohydrate vegetables and including ground flax seeds in some of your recipes. Some find that a laxative such as Movicol or equivalent may act as a helpful regulator in the early stages while their system adjusts.**

***This may be a very important therapeutic component of ketogenic therapy. Research is underway.**

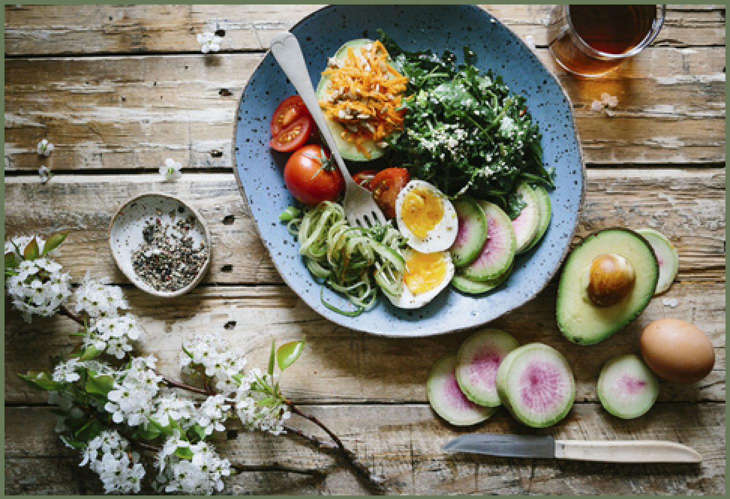

## Weight loss or gain?

It is possible to lose, maintain or even gain weight on ketogenic therapy.

Unplanned weight loss will occur if you are not managing to eat all your required fat portions. Once your body is switched into fat burning mode, it will burn the fats you eat or stored body fats if your food fat intake is too low. Therefore, if you are not consuming enough fats, you may lose weight, feel tired and ketone production may be impaired or exaggerated depending on your individual response.
Unplanned weight gain is not common but may occur if too much fat and protein is eaten and indicates that your fuel intake is greater than your needs. When your weight is increasing, you will not produce ketones as effectively, leading to lower levels.

# Other common queries

### What do I do if I am following the Modified Ketogenic diet and I become unwell?

You will be susceptible to the same illnesses as anyone else and will be given guidance on managing routine illness such as diarrhoea, vomiting, colds etc.

### Will I be able to eat out on the Modified Ketogenic diet?

There are more keto–friendly options on pub and restaurant menus these days so the answer is a definite, yes. However, you are likely to need a little time to build up your own meal planning and carbohydrate counting skills before you can step into a restaurant with confidence.

The Matthew's Friends Handy Guide to Eating Out (available to download from the website or can be sent to you) can provide you with some helpful tips.

https://www.matthewsfriends.org/wp-content/uploads/2015/09/Handy-Guide-to-Eating-Out-June-2015.pdf

### Can I use commercial low carbohydrate food products such as snack bars / cakes etc?

During the initial three-month ketogenic trial phase, it is best to base your meals on simple whole fresh foods.

Some products specifically designed for the Atkins diet contain sugar alcohols such as xylitol and erythritol and sorbitol. Although acceptable for use on the Atkins Diet, they are not recommended to be included in the Modified Ketogenic Diet in the initial stages.

Once you are well tuned-in to any symptom changes the diet has delivered, you may wish to experiment with the occasional low-carb alternative.

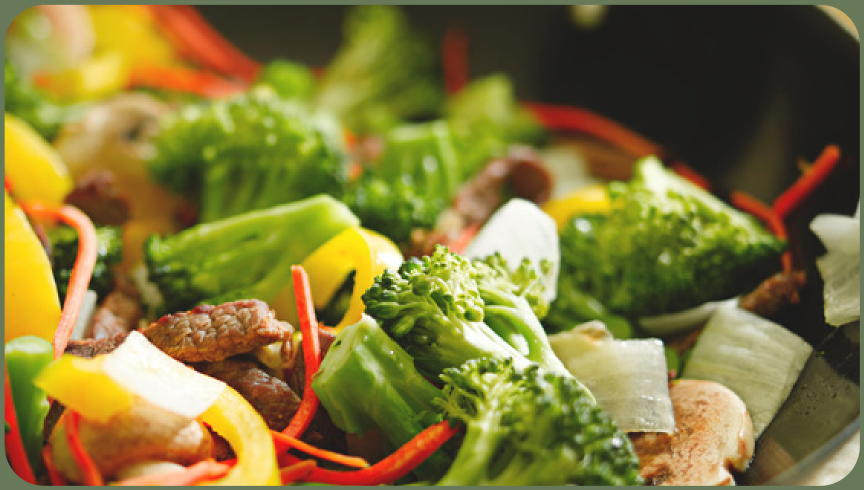

# Eating for improved health & wellbeing;

## Protein Choices

- Animal sources such as meats, fish (white and oily), eggs, cheese, milk,
- Vegetable sources such as beans, lentils, nuts and seeds.
- Most of us eat sufficient protein but perhaps limit our range of sources. Try to include a protein choice at each meal.

## Carbohydrate Choices from:

- Vegetables and fruits. Most of us have too few of these. Go for generous portions vegetables and salads with moderate amounts whole fruits (e.g non-starchy green leafy vegetables and low-sugar fruits e.g berries). Choose a rainbow of colours for nutrient value and eye appeal.
- Grains and white potatoes will need to be limited or find low carbohydrate alternatives e.g.  low carb bread.

## Fats and Oils from:

- Vegetable sources such as olive oil (high in monounsaturated fats; a good main choice), other nut and seed oils (high in polyunsaturated fats; use in small amounts), whole or ground nuts and seeds, avocados.
- Animal sources such as butter, egg yolks, oily fish, meats.
- We all need to include some good quality fats and oils in our meals, they are essential for health.

**The following meal-building suggestions may start you thinking of new food combinations you may like to try.  Just keep things simple and explore changes, one at a time.**

NOTE: If you are a diabetic on insulin or taking medicationsYour medicine doses may need adjustment.

| | |
|---|---|
| **Breakfast** | • Choose a good **Protein Choice** e.g. eggs, beans, bacon, ham, mackerel or nut butters.<br>• Include **Vegetable Choices** such as tomatoes, mushrooms or salads to give colour, crunch and a nutrient boost. Cook or dress these with your favourite oils.<br>• Include a moderate portion of a **Lower GI Carbohydrate Choice** such as low carb bread.<br>OR<br>• For a cereal option, try a Lower GI Carbohydrate Choice based on wholegrain oats e.g. small amount of porridge or greek yoghurt.<br>• Moderate the cereal portion and boost the protein, fat and nutrient value by adding nuts, seeds, unsweetened yogurt or milk. Add moderate portions of fruit (eg berries) to sweeten and boost nutrient variety further. |
| **Meal-Mix** | • Vary your **Protein Choice** e.g. meats or white fish or oily fish or beans or eggs or cheese<br>• Add **Vegetable Choices**. Experiment with colour variety and texture; crunchy vegetable sticks, rainbow salads, chunky vegetable soups, stir fries and roasted vegetable mixes.  Cook or dress these with your favourite oils.<br>• Add a **Lower GI Carbohydrate Choice** e.g. pasta, noodles, brown basmati rice, sweet potatoes, wholegrain granary / seedy type or stoneground wholemeal or sourdough breads or oatcakes.<br>• **Dessert:**  choose fresh fruit and whole milk yogurts more often |
| **Snack Mix** | • Fresh fruits including berries<br>• Nuts and seeds<br>• Yogurts (preferably whole milk based and unsweetened)<br>• Oatcakes with cheese<br>• Vegetable sticks with houmous, yogurt based dips or pate<br>• Plain dark chocolate - in moderation |
| **Drinks** | • Water (Add a citrus slice or cucumber and mint leaves to ring the changes).<br>• Tea, coffee, herbal / fruit teas (no added sugar)<br>• Small amounts of unsweetened milk (dairy, soya, nut milks etc.)<br>• Sugar free flavoured squashes / waters etc. |
